# Effect of co-infection with a small intestine-restricted helminth pathogen on oral prion disease pathogenesis in mice

**DOI:** 10.1038/s41598-019-42900-9

**Published:** 2019-04-30

**Authors:** Alejandra Sánchez-Quintero, Barry M. Bradford, Rick Maizels, David S. Donaldson, Neil A. Mabbott

**Affiliations:** 1 The Roslin Institute & Royal (Dick) School of Veterinary Studies, University of Edinburgh, Easter Bush, EH25 9RG United Kingdom; 2 Wellcome Trust Centre for Molecular Parasitology, Institute for Infection, Immunity and Inflammation, Sir Graeme Davies Building, 120 University Place, Glasgow, G12 8TA United Kingdom

**Keywords:** Neuroimmunology, Mucosal immunology, Infectious diseases

## Abstract

The early replication of some orally-acquired prion strains upon stromal-derived follicular dendritic cells (FDC) within the small intestinal Peyer’s patches is essential to establish host infection, and for the disease to efficiently spread to the brain. Factors that influence the early accumulation of prions in Peyer’s patches can directly influence disease pathogenesis. The host’s immune response to a gastrointestinal helminth infection can alter susceptibility to co-infection with certain pathogenic bacteria and viruses. Here we used the natural mouse small intestine-restricted helminth pathogen *Heligmosomoides polygyrus* to test the hypothesis that pathology specifically within the small intestine caused by a helminth co-infection would influence oral prion disease pathogenesis. When mice were co-infected with prions on d 8 after *H. polygyrus* infection the early accumulation of prions within Peyer’s patches was reduced and survival times significantly extended. Natural prion susceptible hosts such as sheep, deer and cattle are regularly exposed to gastrointestinal helminth parasites. Our data suggest that co-infections with small intestine-restricted helminth pathogens may be important factors that influence oral prion disease pathogenesis.

## Introduction

Prion infections cause chronic neurodegenerative diseases in humans and animals for which there are no treatments. Aggregations of PrP^Sc^, abnormally folded isoforms of the host-encoded cellular prion protein (PrP^C^), accumulate in the affected tissues of prion disease infected hosts and are considered to constitute the major component of the infectious agent^1–3^. Many prion strains such as natural sheep scrapie, bovine spongiform encephalopathy (BSE) in cattle and chronic wasting disease (CWD) in cervid species can be orally acquired. Furthermore, some prion strains can have zoonotic potential as the consumption of BSE-contaminated food was responsible for the subsequent emergence of variant Creutzfeldt-Jakob disease (vCJD) in the UK human population^4^. Data from experimental mice^5^, sheep with natural scrapie^6^, deer with CWD^7^ and vCJD patients^8^ show that some orally acquired prion strains replicate first upon stromal-derived follicular dendritic cells (FDC) in the gut-associated lymphoid tissues (GALT) as they make their journey from the gut to the brain (termed *neuroinvasion*). Mouse studies show that the initial replication of the prions within the GALT is essential to establish host infection^5,9–12^. Prions then spread from the GALT via the enteric and peripheral nervous systems to the brain where there accumulation ultimately leads to neurodegeneration and death^13,14^.

The incidence of clinical cases of vCJD in humans has fortunately been much lower than originally estimated despite the probable widespread exposure of the UK population to BSE-contaminated food in the 1980s. This implies that factors in addition to host *PRNP* genotype (which encodes PrP^C^) and prion agent strain can also influence an individual’s susceptibility to oral prion infection. For example, the adverse effects of ageing on the GALT microarchitecture impede the early uptake and replication of orally-acquired prions within these tissues and reduce disease susceptibility^15,16^. This may help to explain why the majority of the clinical vCJD cases have been predominantly recorded in young individuals (typical median age at onset of clinical disease ~26 years old)^17,18^.

Pathogen induced alterations to GALT also have the potential to influence prion uptake and disease susceptibility. For example, orally-acquired prions are initially transported across the gut lumen into Peyer’s patches by a specialized population of phagocytic epithelial cells known as M cells^19–21^. Oral prion disease is blocked in mice lacking these cells^21,22^, and exacerbated in mice with an increased M cell-density^22^. Oral infection with certain pathogenic bacteria or exposure to inflammatory stimuli such as cholera toxin can increase M-cell density in the intestine^23–25^, and with the damage and/or inflammation caused by pathogen infection^26^ this could potentially exacerbate oral prion disease pathogenesis by increasing the efficiency of the initial uptake of the prions from the gut lumen. Mononuclear phagocytes (MNP) are a diverse population of macrophages and classical dendritic cells (DC)^27–29^ that also play important and contrasting roles during prion disease depending on the subset^30^. Whereas CD11c^+^ classical DC aid the propagation of orally acquired prions towards FDC in Peyer’s patches in order to establish infection^31,32^, the uptake of prions by macrophages can lead to their destruction^33,34^. Therefore, pathogen-induced alterations to the abundance, trafficking or activity of MNP could similarly influence disease pathogenesis by enhancing the propagation or clearance of orally-acquired prions.

Studies in mice have shown that the host’s immune response to a gastrointestinal helminth infection alters susceptibility to co-infection with a variety of other pathogenic microorganisms^35^ including *Salmonella enterica* serovar Typhimurium^36^, *Citrobacter rodentium*^37^ and norovirus^38^. Natural prion susceptible hosts such as sheep, deer and cattle are regularly exposed to gastrointestinal helminth parasites, but little is known of the effects that co-infection with these pathogens may have on oral prion disease pathogenesis. Since neuroinvasion of orally-acquired prions occurs directly from the GALT in the small intestine^12^, in this study the natural mouse small intestine-restricted helminth pathogen *Heligmosomoides polygyrus* was selected. This parasite is an excellent model for the study of gastrointestinal helminth infections in livestock and humans^39^, being phylogenetically similar to the ruminant parasites *Haemonchus contortus* and *Teladorsagia circumcincta*, and human hookworms *Ancylostoma duodenale* and *Necator americanus*^40^. We used *H. polygyrus* to test the hypothesis that the pathology caused by a pathogen co-infection specifically within the small intestine would significantly influence oral prion disease pathogenesis. These data are essential for the identification of important factors can that influence the risk of oral prion disease transmission and to help design effective intervention and control strategies.

## Results

### Oral *H. polygyrus* infection causes pathology in the small intestine

**Figure 1:**
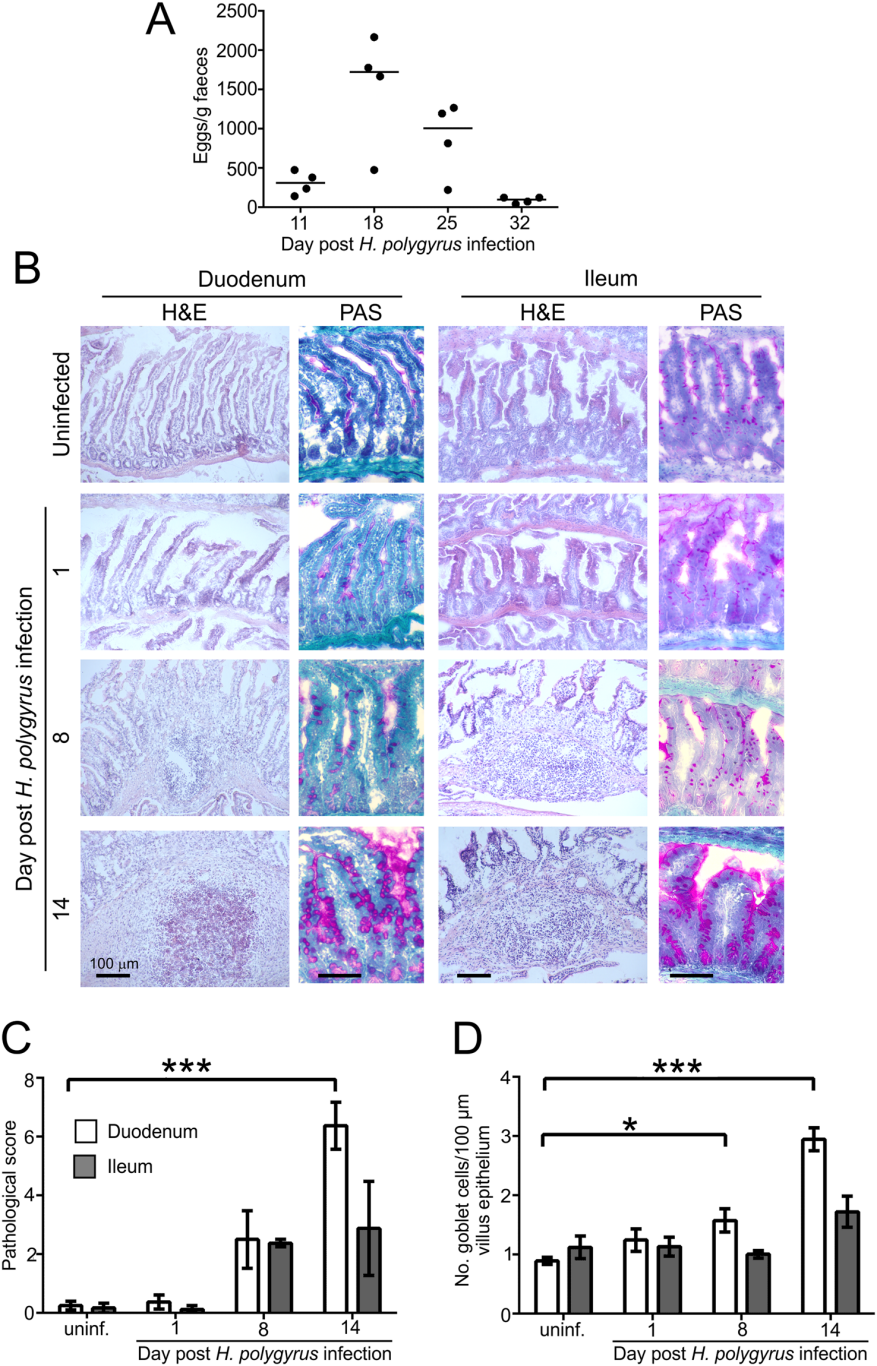
Oral *H. polygyrus* infection causes pathology in the small intestine. (**A**) Faecal egg burdens following oral infection of C57BL/6J mice with 200 *H. polygyrus* L3 larvae by gavage. Horizontal bar, median. (**B**) Microscopical analysis of the effects of *H. polygyrus* infection on the small intestine. Mice were orally infected with *H. polygyrus* and sections of the duodenum and ileum collected at intervals afterwards and stained with haematoxylin and eosin (H&E) or PAS to detect mucous-secreting goblet cells (pink). (**C**) Morphometric assessment of the magnitude of the histopathology within the duodenum and ileum at intervals after *H. polygyrus* infection. 4 blinded non-sequential sections of duodenum and ileum per mouse were scored for inflammatory cell infiltrate (0–4) and alterations to the villus architecture (0–4). Mean scores per mouse were combined to generate the final score (0–8). ***P < 0.0001, Two-way ANOVA. (**D**) Goblet cell density in the epithelium of the duodenum was significantly increased on d 8 and d 14 after *H. polygyrus* infection. *P < 0.01; ***P < 0.0001, Two-way ANOVA. Data are derived from 16–40 villi across 2–3 images/mouse, *n* = 3–4 mice/group. Error bars show SEM.

Groups of four female C57BL/6J mice were orally infected with 200 *H. polygyrus* L3 larvae by gavage and faecal egg burdens measured at intervals afterwards to monitor the magnitude of the parasite infection. As anticipated, maximum egg production was observed by 18 days post-infection (dpi) with *H. polygyrus* and had declined by 32 dpi (Fig. 1A). Histopathological analysis of inflammatory cell infiltrate and changes to the intestinal architecture (using the evaluation scheme described in ref.^41^) confirmed the presence of detectable pathology within the small intestine by 8 dpi with *H. polygyrus*, and was significantly greater in the duodenum by 14 dpi (Fig. 1B,C). Goblet cell density in epithelium of the duodenum was also significantly increased from 8 dpi with *H. polygyrus* (Fig. 1D).

**Figure 2:**
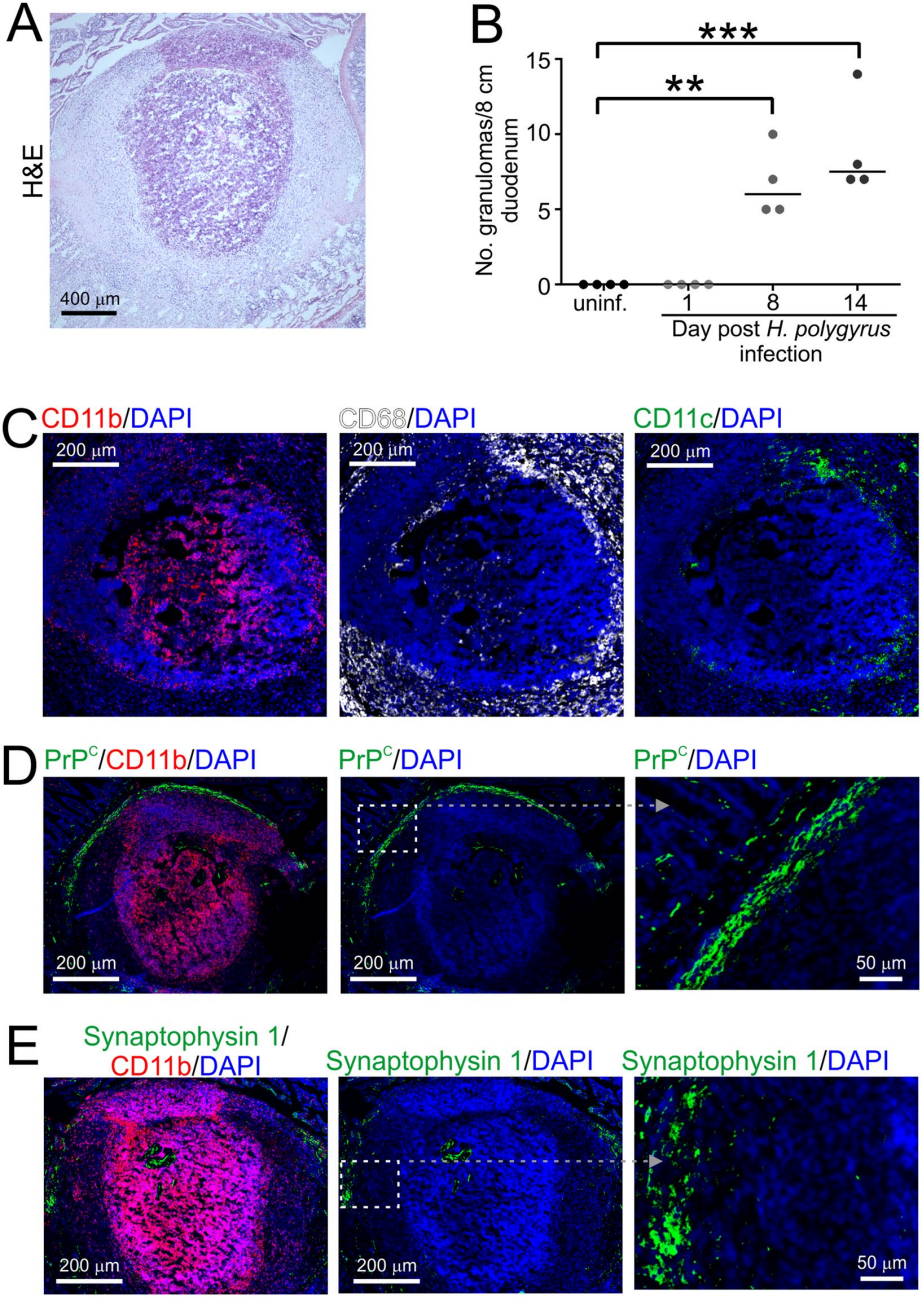
Oral *H. polygyrus* infection induces the formation of granulomas in the mucosa of the small intestine. (**A**) Numerous macroscopic granulomas were evident in the duodenal wall from 8 d after *H. polygyrus* infection (H&E, haematoxylin and eosin-stained section). (**B**) Numeration of the number of visible granulomas in two non-sequential 4 cm sections of duodenum. Each point represents data from an individual mouse. Horizontal bar, median of 4 mice/group. **P < 0.003; ***P < 0.0002, One-way ANOVA. (**C**) Immunohistochemical (IHC) analysis showed that the granulomas comprised a central core predominantly containing CD11b^+^ (red) mononuclear phagocytes (MNP), with an outer ring containing CD68^+^ MNP (white) and CD11c^+^ MNP (green). (**D,E**) Granulomas were typically surrounded by a border of PrP^C^-expressing stromal-like cells (**D**, green) and cells expressing the neuronal synaptic vesicle marker synaptophysin 1 (**E**, green) indicative of enteric nerves. (**C**–**E**) sections were counterstained with DAPI (blue) to detect cell nuclei. Boxed regions in (**D,E**) are shown at higher magnification in the adjacent right-hand panels.

Numerous macroscopic granulomas were also evident in the duodenal wall from 8 dpi with *H. polygyrus* infection (Fig. 2A,B). Consistent with previous reports^42^ our immunohistochemical (IHC) analysis showed that these structures typically comprised a central core which predominantly contained CD11b^+^ MNP, surrounded by other populations of CD68^+^ and CD11c^+^ MNP (Fig. 2C). Independent studies have suggested that an inflammatory PrP^C^-expressing stromal cell population within soft tissue granulomas can support prion replication^43^. IHC analysis did not reveal significant cell-associated PrP^C^ expression within the granulomas induced in the gut wall during *H. polygyrus* infection. However, they were typically surrounded by a border of PrP^C^-expressing stromal-like cells (Fig. 2D), as well as cells expressing the neuronal synaptic vesicle marker synaptophysin 1, indicative of enteric nerves (Fig. 2E), suggesting a potential novel site for prion accumulation or neuroinvasion in the gut wall.

### Effect of *H. polygyrus* co-infection on oral prion disease susceptibility

Next, groups of C57BL/6J mice (*n* = 6–8) were orally-infected with *H. polygyrus* and 0, 1, 8 or 14 d later the mice were subsequently orally co-infected with a limiting dose of ME7 scrapie prions. The days on which the mice were co-infected with prions were selected based on the known pathogenesis of the *H. polygyrus* infection in the small intestine^44^: 1 dpi, when the infective L3 larvae penetrate the lining of the duodenum; 8 dpi, during the period when the adult worms re-emerge back into the gut lumen; 14 dpi, when adult worms are tethered around the villi in the duodenum. A separate group of mice were orally-infected with prions alone as a control.

**Figure 3:**
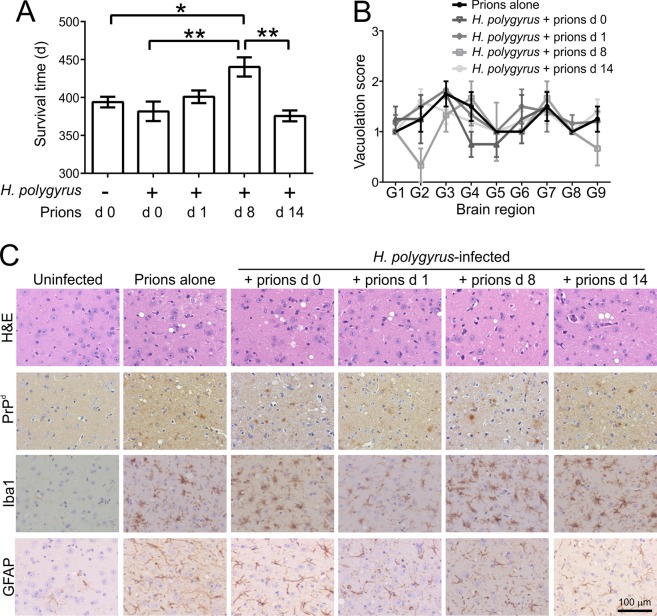
Effect of *H. polygyrus* co-infection on oral prion disease. Groups of 6–8 C57BL/6J mice were orally-exposed to a limiting dose of ME7 scrapie prions on either d 0, 1, 8 or 14 after oral infection with *H. polygyrus* L3 larvae. A separate group of mice were exposed to prions alone as a control. (**A**) Mean prion disease survival times (±SEM) for the orally-infected mice from each group. When the mice were co-infected with prions on d 8 post-infection with *H. polygyrus* infection, the mean survival times were significantly extended by approximately 39 days when compared to mice infected with prions alone. *n* = 3–6 clinically positive mice/group *P < 0.05; **P < 0.01, One-way ANOVA. (**B**) High levels of spongiform pathology (H&E, upper row), heavy accumulations of PrP^d^ (brown, second row), active microglia expressing Iba-1 (brown, third row) and reactive astrocytes expressing GFAP (brown, bottom row) were detected in the brains of all the orally-exposed mice with clinical prion disease at the time of cull. Sections were counterstained with haematoxylin to detect cell nuclei (blue). Images show the thalamus. (**C**) The severity and distribution of the spongiform pathology (vacuolation) within each clinically-affected brain was scored on a scale of 1–5 in nine grey matter regions: G1, dorsal medulla; G2, cerebellar cortex; G3, superior colliculus; G4, hypothalamus; G5, thalamus; G6, hippocampus; G7, septum; G8, retrosplenial and adjacent motor cortex; G9, cingulate and adjacent motor cortex; Each point represents the mean vacuolation score ± SEM, *n* = 3–6 clinically positive mice/group, not significantly different to Prions alone, Two-way ANOVA.

Oral exposure of C57BL/6 mice to a limiting dose of ME7 scrapie prions has an incomplete clinical disease incidence^22,32^, allowing the potential effects of *H. polygyrus* co-infection on survival times and disease susceptibility to be determined. As expected, four of eight of the mice exposed to prions alone developed clinical disease with a mean survival time of 394 ± 14 d (median = 401 d; Fig. 3A, Table 1). The remaining mice in this group did not develop clinical prion disease up to at least 504 d after exposure. The mice co-infected with prions on d 0, 1 and 14 after *H. polygyrus* infection also succumbed to clinical prion disease with similar survival times and disease incidences to those infected with prions alone (Fig. 3A, Table 1). However, when the mice were co-infected with prions on d 8 after *H. polygyrus* infection their mean survival time was significantly extended by approximately 39 d (mean = 440 d, median = 436 d, *n* = 3/6; Fig. 3A, Table 1).

**Table 1:** Effect of *H. polygyrus* co-infection on oral prion disease pathogenesis.

Mouse model^a^	Mean survival times^b^ (individual durations, days)^c^	Median survival times (days)	Clinical disease^d^	Spongiform pathology in the brain^e^	*P* value^f^
Prions alone	394 ± 14 (373, 401, 401, 401; 4X > *504*)	401	4/8	4/8	
*H. polygyrus* + prions d 0	380 ± 12 (352, 380, 380, 415; 1X > *489*, 3X > *504*)	380	4/8	4/8	NS
*H. polygyrus* + prions d 1	401 ± 20 (373, 394, 401, 401, 401, 436; 1X > *504*)	401	6/7	6/7	NS
*H. polygyrus* + prions d8	440 ± 22 (421, 436, 464; 1X *496*, 2X > *504*)	436	3/6	3/6	*
*H. polygyrus* + prions d 14	376 ± 16 (352, 366, 387, 387, 387; 1X > *504*)	387	5/6	5/6	NS

^a^C57BL/6J mice were orally-exposed to a limiting dose of ME7 scrapie prions on either d 0, 1, 8 or 14 after oral infection with 200 *H. polygyrus* L3 larvae.

^b^Mean duration (±SD) from time of injection with prions to cull at clinical end-point.

^c^Prion disease durations for individual mice (from time of injection with prions to cull at clinical end-point). The notation “NX > *504*” means mice that were free of the clinical and histopathological signs of prion disease up to at least this time after oral exposure.

^d^Incidence = no. animals displaying clinical signs of prion disease/no. animals tested.

^e^Incidence = no. animals with histopathological signs of prion disease in the brain (spongiform pathology)/no. animals tested.

^f^Statistical differences between survival times were compared by one-way ANOVA with Tukey’s post hoc test. *P < 0.05, when compared to mice orally-exposed to prions alone. NS, not significant.

All the brains from the mice in each infection group that developed clinical signs of prion disease displayed the characteristic spongiform pathology (neuronal vacuolation), microgliosis, astrogliosis and accumulations of prion disease-specific PrP (referred to as PrP^d^) that are associated with a terminal, clinical, infection with ME7 scrapie prions (Fig. 3B**)**. The distribution and severity of the spongiform pathology was also similar in the brains of the mice that developed clinical signs of prion disease (Fig. 3C). None of these histopathological signs of prion disease were detected in any of the brains from the clinically-negative mice (data not shown). Thus although co-infection with prions on d 8 after *H. polygyrus* infection significantly extended the survival times, the parasite co-infection did not alter the course of CNS prion disease once neuroinvasion had occurred.

### Effect of *H. polygyrus* co-infection on the early accumulation of prions in Peyer’s patches and the MLN

**Figure 4:**
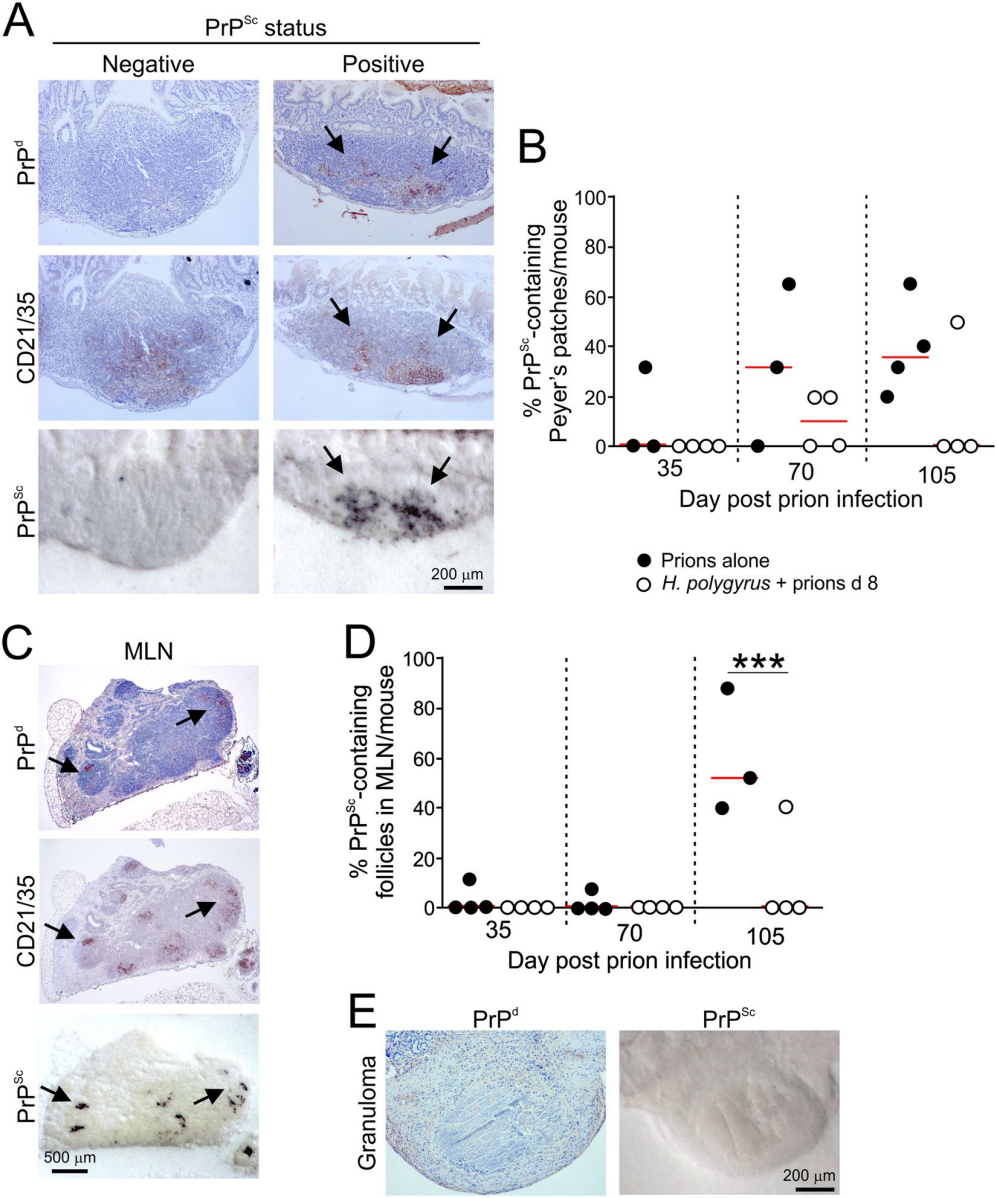
Effect of *H. polygyrus* co-infection on the accumulation of prions upon FDC in Peyer’s patches and the MLN. C57BL/6J mice were orally-infected with *H. polygyrus* L3 larvae and subsequently orally exposed to a limiting dose of ME7 scrapie prions 8 d later. A separate group of mice were exposed to prions alone as a control. (**A**) Right-hand panels show the typical histopathological presentation of a PrP^Sc^-positive Peyer’s patch after oral prion exposure. High levels of prion disease-specific PrP (PrP^d^, brown, upper row) were detected in association with FDC (CD21/35^+^ cells, brown, middle row, arrows), and PET immunoblot analysis confirmed the presence of PK-resistant PrP^Sc^ (blue/black, bottom row). PrP^Sc^-negative Peyer’s patches lacked these accumulations (left-hand panels). (**B**) The frequency of PrP^Sc^-positive Peyer’s patches in the small intestines of mice given prions alone or 8 d after *H. polygyrus* infection at intervals after oral prion exposure (*n = *3–4 mice/group). The frequency of PrP^Sc^-containing Peyer’s patches was lower in mice co-infected with prions on d 8 after *H. polygrus* infection. Horizontal bars, median. (**C**) Typical histopathological images showing the accumulation of high levels of prion disease-specific PrP^Sc^ in association with FDC (arrows) in the MLN after oral prion infection. (**D**) The frequency of PrP^Sc^-positive FDC in the MLN of mice given prions alone or 8 d after *H. polygyrus* infection at intervals after oral prion exposure (*n = *3–4 mice/group). Horizontal bars, median. Significant differences in (**B and C**) were determined by two-way ANOVA. ***P < 0.001. (**E**) Although granulomas were present in the small intestines of many of the co-infected mice, no histopathological evidence of PrP^d^ or PrP^Sc^ accumulation was detected within them (20 granulomas assessed across different time points).

The early replication of ME7 scrapie prions in association with FDC in the Peyer’s patches is essential to establish host infection and for their efficient spread to the CNS^5,10,12^, and factors that impede this can extend survival times^5,21,22,31,32^. We next determined whether the early accumulation of PrP^Sc^ upon FDC in the Peyer’s patches was impeded in mice co-infected with prions on d 8 after *H. polygyrus* infection. In mice infected with prions alone, FDC-associated (CD21/35^+^ cells) PrP^Sc^ accumulations were first detected in a small percentage of the Peyer’s patches by 35 dpi and had increased in frequency by 105 dpi (Fig. 4A,B). However, the frequency of PrP^Sc^-containing Peyer’s patches was reduced in mice co-infected with prions on d 8 after *H. polygyrus* infection at each of the time points analysed (Fig. 4B).

Within weeks after their initial replication within Peyer’s patches, the prions them disseminate to the MLN and most other secondary lymphoid tissues^5,10,12,21,31^ (Fig. 4C,D). The frequency of PrP^Sc^-positive FDC networks in the MLN of the mice co-infected with prions on d 8 post-*H. polygyrus* infection was similarly reduced (Fig. 4D). Although granulomas were present in the small intestines of many of the co-infected mice, no histopathological evidence of PrP^Sc^ accumulation was detected within them (Fig. 4E).

Our data suggested that specific pathological effects on the small intestine at 8 dpi with *H. polygyrus* had impeded the initial accumulation of prions upon FDC in Peyer’s patches. Further experiments were undertaken to attempt to identify the pathological factors that mediated this effect.

### *H. polygyrus* infection does not affect the density and function of M cells

**Figure 5:**
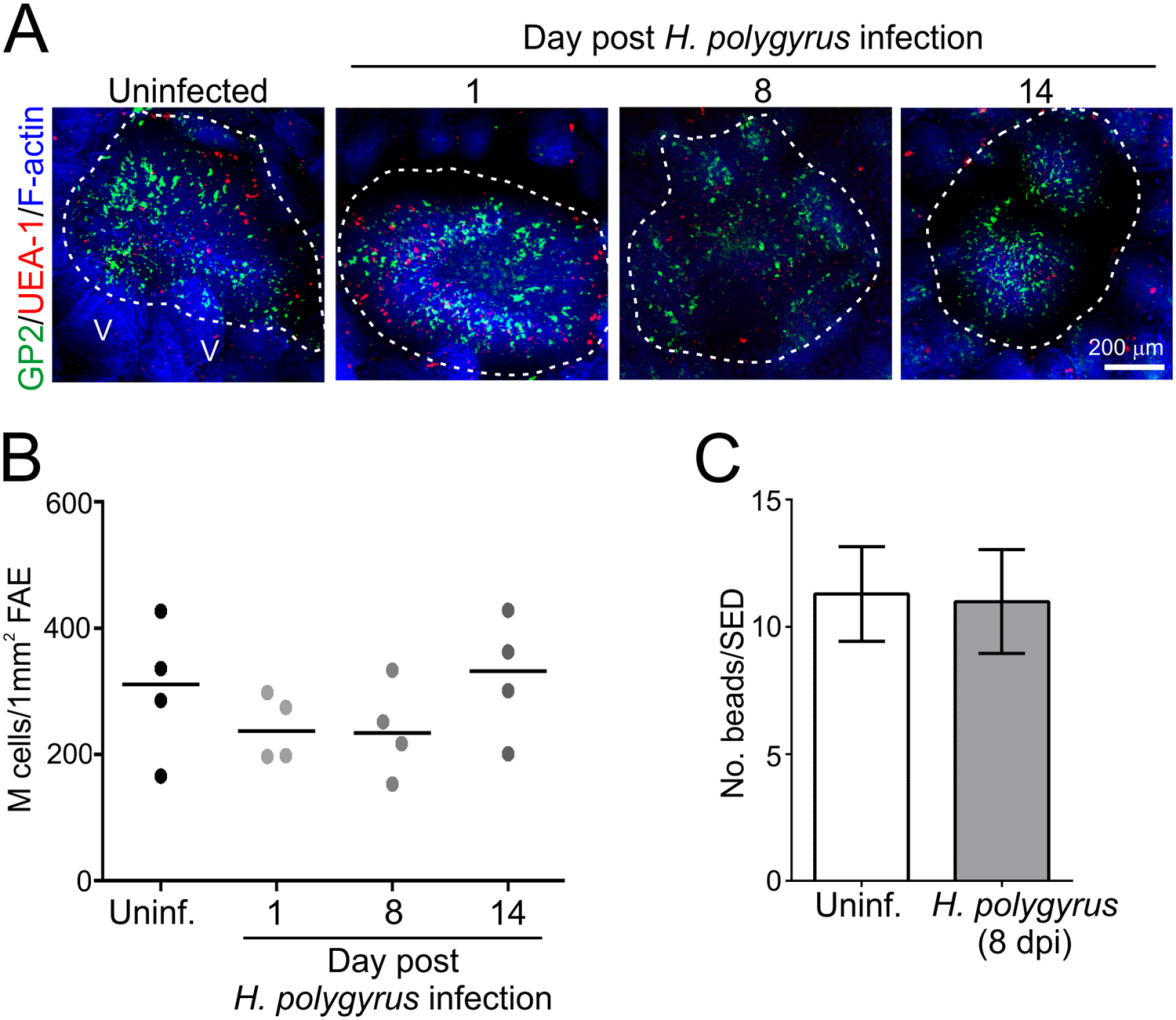
*H. polygyrus* infection does not influence M cell status in the small intestine. C57BL/6J mice were orally infected with 200 *H. polygyrus* L3 larvae and Peyer’s patches collected 1, 8 and 14 d later. Uninfected mice were used as controls. (**A**) Peyer’s patches were whole-mount immunostained to detect M cells (GP2^+^ cells, green) and goblet cells (UEA-1 binding cells, red). Phalloidin was used to detect F-actin (blue). The broken lines indicate the boundary of the follicle-associated epithelium (FAE). V, villi. (**B**) Morphometric analysis indicated that density of GP2^+^ M cells in the FAE was similar in the Peyer’s patches from each mouse group (data derived from 2 follicles/Peyer’s patch, 1–2 Peyer’s patches/mouse, *n* = 4, not significant, One-way ANOVA). (**C**) C57BL/6J mice were orally infected with 200 *H. polygyrus* L3 larvae and 5 d later were orally gavaged with 200 nm fluorescent microbeads. Uninfected (Uninf.) mice were orally-gavaged with microbeads as a control. Seventy two hours later the number of microbeads which had been transcytosed into the subepithelial dome region of their Peyer’s patches was quantified on tissue sections by fluorescence microscopy. Similar levels of fluorescent microbeads were detected in the subepithelial dome regions of the Peyer’s patches from mice from each group. Data were collected from 32–33 subepithelial dome regions across 6–12 Peyer’s patch sections from 4 mice/group, not significant, Mann-Whitney U test.

Orally-acquired prions are initially transferred across the intestinal epithelium into Peyer’s patches by M cells, and this is essential to establish infection^21^. Furthermore, changes to M cell-density in the specialized follicle-associated epithelium (FAE) that covers the Peyer’s patches can directly influence oral prion disease susceptibility^22^. Whole-mount IHC showed that the density of M cells expressing the mature M-cell marker glycoprotein 2 (GP2; refs.^45,46^) in FAE covering the Peyer’s patches was unchanged after *H. polygyrus* infection (Fig. 5A,B). The *in vivo* ability of M cells to acquire and transcytose particulate antigens can be assessed by measuring the uptake of fluorescently-labelled latex microbeads from the gut lumen into Peyer’s patches^22,45,47,48^. Here, C57BL/6J mice (*n* = 4) were orally infected with *H. polygyrus* and at 5 dpi orally-gavaged with fluorescent microbeads. Uninfected mice were orally-administered fluorescent microbeads as a control. Seventy two hours later (d 8 post-*H. polygyrus* infection) the number of microbeads that had been transcytosed into the subepithelial dome (SED) region of their Peyer’s patches was quantified on tissue sections by fluorescence microscopy. Similar levels of fluorescent microbeads were detected in the SED regions of the Peyer’s patches from mice from each group (Fig. 5C). These data show that the density of M cells in the FAE, and their ability to transcytose particulate antigens from the gut lumen into Peyer’s patches, was not impaired following *H. polygyrus* infection.

### Effects of *H. polygyrus* infection on FDC in Peyer’s patches and the MLN

**Figure 6:**
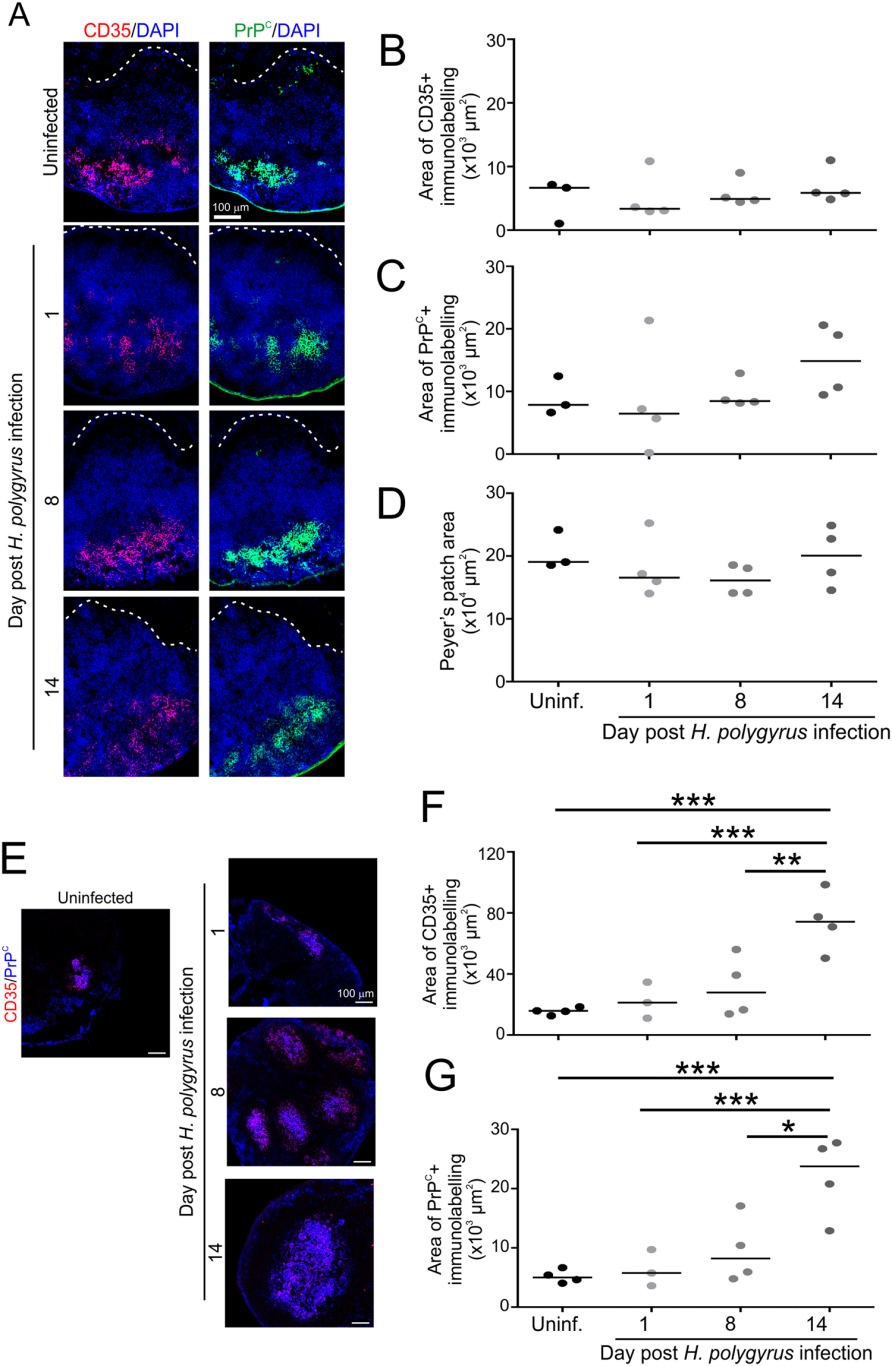
Effect of *H. polygyrus* infection on follicular dendritic cell status in Peyer’s patches and the MLN. C57BL/6J mice were orally infected with 200 *H. polygyrus* L3 larvae and Peyer’s patches collected 1, 8 and 14 d later. Uninfected (Uninf.) mice were used as a control. (**A**) Immunohistochemical comparison of CD35 (red, upper row) and PrP^C^ (green, bottom row) expression by follicular dendritic cells (FDC) in Peyer’s patches following *H. polygyrus* infection. Cell nuclei were counterstained with DAPI (blue). (**B**) Morphometric analysis revealed that the area of the CD35^+^ FDC networks in Peyer’s patches from mice from each group were similar. (**C**) The % area of PrP^C^ immunostaining within the area of the FDC networks was also similar in Peyer’s patches from each group. (**D**) The overall area of the Peyer’s patches also similar in the intestines of mice from each group. Horizontal bars, median. Data derived from 1–2 Peyer’s patch follicles per mouse, *n = *3–4 mice/group. (**E**) Immunohistochemical comparison of CD35 (red) and PrP^C^ (blue) expression by FDC in the MLN following *H. polygyrus* infection. Morphometric analysis of the area of the (**F**) CD35^+^ and (**G**) PrP^C^-positive immunolabelling in MLN from mice from each group revealed a significant increase on d 14 post *H. polygyrus* infection when compared to uninfected controls (*P < 0.05; **P < 0.01; ***P < 0.007; one-way ANOVA). Horizontal bars, median. Data derived from 1–7 fields of view (images) per mouse, *n = *3–4 mice/group.

FDC initially retain complement-opsonized prions on their surfaces in a complement receptor-dependent (CR) manner, and CR1 (CD35) is considered to play a prominent role^49^. IHC and morphometric analysis showed that the FDC were similar in size in the Peyer’s patches from *H. polygyrus*-infected and uninfected control mice (area of CD35^+^ immunostaining; Fig. 6A,B). The expression of PrP^C^ by FDC is essential to sustain prion replication upon their surface^14,50^. Our analysis also showed that the magnitude of the PrP^C^-expression co-localized upon FDC was similar in Peyer’s patches from each group (Fig. 6A,C). The overall area of the Peyer’s patches was also unchanged during *H. polygyrus*-infection (Fig. 6D). In contrast, in the MLN a significant increase in the size of the FDC and the expression of PrP^C^ was observed on d 14 post *H. polygyrus* infection when compared to uninfected control mice (Fig. 6E–G). These data clearly show that the delayed accumulation of PrP^Sc^ in the Peyer’s patches and MLN of mice infected with prions on d 8 post *H. polygyrus* infection was not simply due to reduced FDC size or PrP^C^ expression.

### Effects of *H. polygyrus* infection on the MNP in the lamina propria

**Figure 7:**
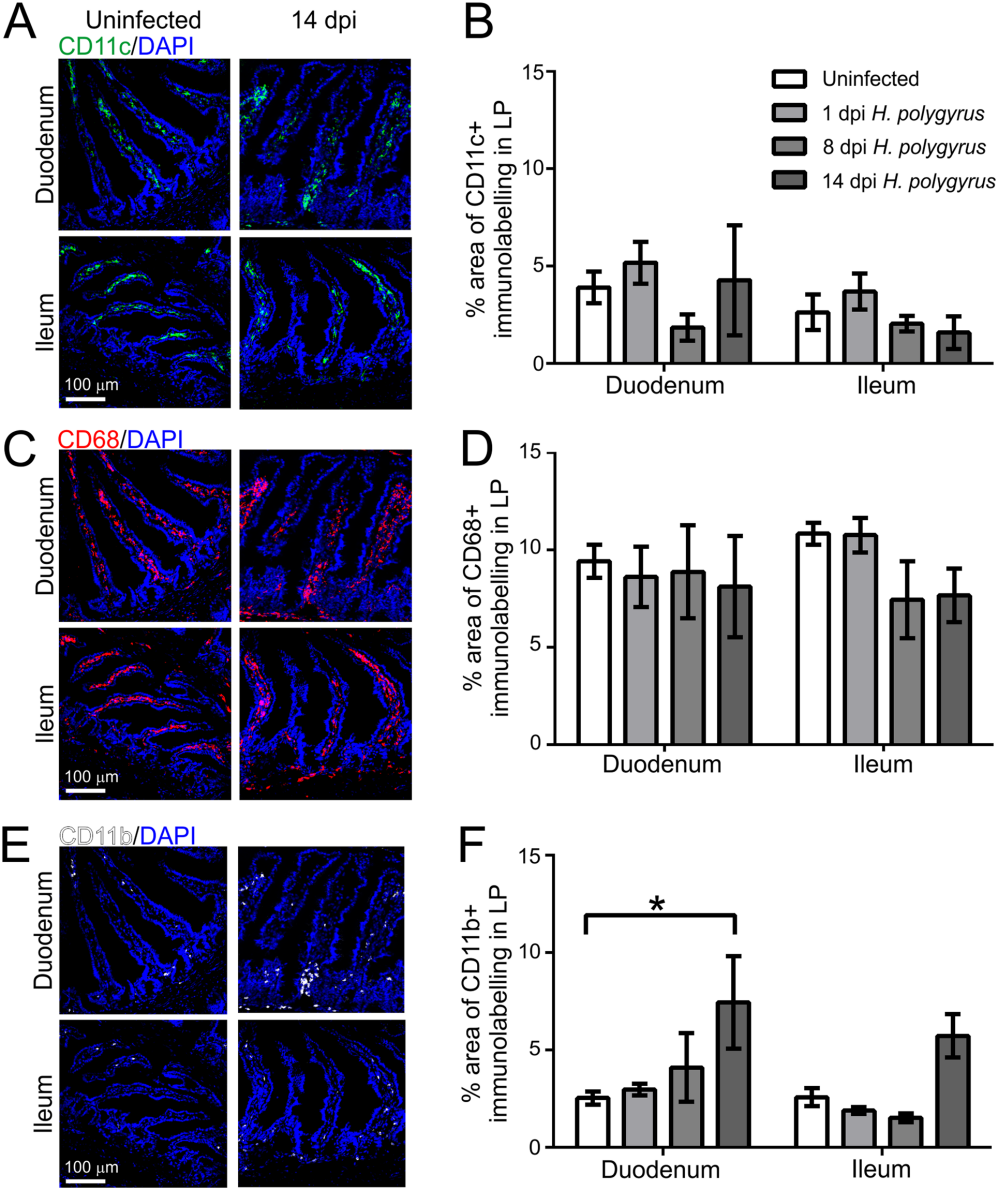
Effect of *H. polygyrus* infection on the abundance of mononuclear phagocytes in the lamina propria of the small intestine. C57BL/6J mice were orally infected with 200 *H. polygyrus* L3 larvae and intestines collected 1, 8 and 14 d later. Uninfected mice were used as controls. Immunohistochemical comparison of the typical distribution of (**A**) CD11c^+^ (**C**) CD68^+^ and (**E**) CD11b^+^ mononuclear phagocytes in the lamina propria of the duodenum (upper row) and ileum (lower row) in uninfected (control) mice and at 14 d after *H. polygyrus* infection. Sections were counterstained with DAPI to detect cell nuclei (blue). (**B**,**D**,**F**) Morphometric analysis of the number of CD11c^+^, CD68^+^ and CD11b^+^ mononuclear phagocyte in the lamina propria at intervals after *H. polygyrus* infection. A significant increase in the % area of CD11b^+^ immunostaining was observed in the duodenum on d 14 post *H. polygyrus* infection (*P < 0.05, Two-way ANOVA). Data derived from individual lamina propria areas/mouse, *n* = 3–4 mice/group.

Prions are considered to utilize CD11c^+^ MNP to mediate their delivery to FDC^31,32^, whereas macrophages appear to phagocytose and destroy prions^34^. IHC and morphometric analysis suggested that the % area of CD11c^+^ (Fig. 7A,B) and CD68^+^ (Fig. 7C,D) immunostaining in the lamina propria was unchanged in the small intestines of *H. polygyrus* infected mice when compared to uninfected controls. However, a significant increase in the % area of CD11b^+^ immunostaining (Fig. 7E,F) was observed in the duodenum on d 14 post *H. polygyrus* infection, implying an increased abundance of CD11b^+^ MNP in the lamina propria at this later time point.

### Effect of *H. polygyrus* infection on the abundance and distribution of CD11c^+^ MNP in Peyer’s patches

**Figure 8:**
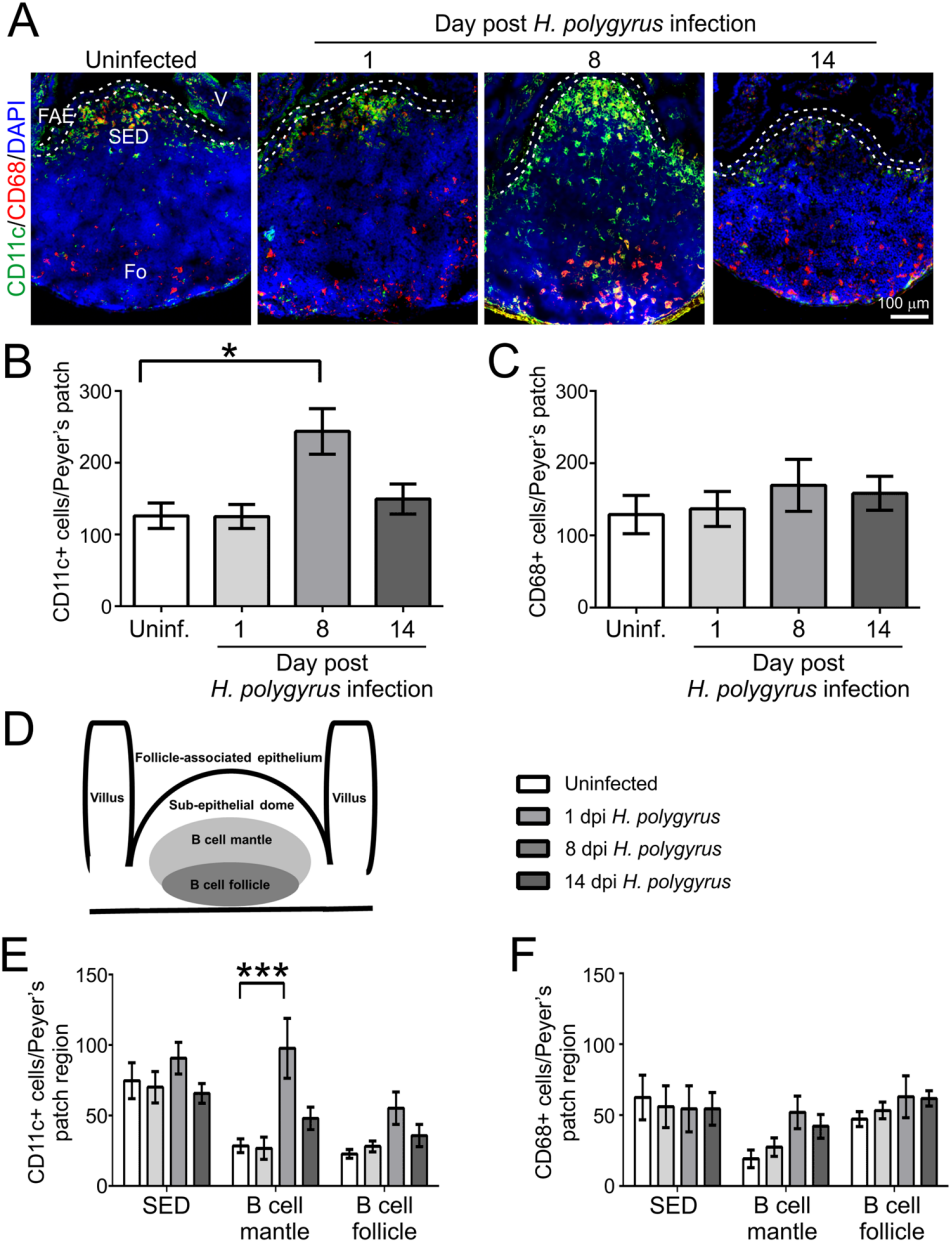
Effects of *H. polygyrus* on the abundance and distribution of mononuclear phagocytes within Peyer’s patches. C57BL/6J mice were orally infected with 200 *H. polygyrus* L3 larvae and Peyer’s patches collected 1, 8 and 14 d later. Uninfected mice were used as controls. (**A**) Immunohistochemical comparison of the typical distribution of CD11c^+^ and CD68^+^ mononuclear phagocytes in the Peyer’s patches at intervals after *H. polygyrus* infection. Cell nuclei were counterstained with DAPI (blue). Broken lines show the boundaries of the follicle-associated epithelium (FAE). Fo, B cell follicle; SED, subepithelial dome; V, villus. (**B**) Morphometric analysis suggested there was a significant increase in the number of CD11c^+^ mononuclear phagocytes in the Peyer’s patches on d 8 post *H. polygyrus* infection (*P < 0.05, One-way ANOVA). (**C**) Morphometric analysis suggested that the number of CD68^+^ mononuclear phagocytes was similar in the Peyer’s patches from mice from each group (not significant, One-way ANOVA). (**D**) Cartoon showing the anatomical regions in the Peyer’s patches that were used for the analysis in panels E and F. (**E**) Morphometric analysis suggested there was a significant increase in the number of CD11c^+^ mononuclear phagocytes in the B cell mantle region of the Peyer’s patches on d 8 post *H. polygyrus* infection (***P < 0.001, Two-way ANOVA). (**F**) Morphometric analysis suggested that the distribution of the CD68^+^ MNP was unchanged after *H. polygyrus* infection (not significant, Two-way ANOVA). Data derived from *n* = 4–7 Peyer’s patches across 3–4 mice/group, and representative of two independent experiments.

After their transcytosis by M cells across the gut epithelium, particulate antigens are released into the M cell’s basolateral pocket where they and subsequently sampled by MNP^29^. We have shown that prions are also subsequently acquired by CD11c^+^ MNP after oral exposure^14,31,51^ and propagated by them towards the FDC in the B cell follicles of Peyer’s patches^32^. IHC and morphometric analysis revealed that the abundance of CD11c^+^ MNP was specifically and significantly increased in the Peyer’s patches on d 8 after *H. polygyrus* infection (Fig. 8A,B). In contrast, the number of CD68^+^ MNP (indicative of tissue macrophages) in the Peyer’s patches was unchanged during *H. polygyrus* infection (Fig. 8A,C). Stratification of the Peyer’s patches into three distinct anatomical regions (SED, B cell mantle and B cell follicle; Fig. 8D) revealed that the distribution of the CD11c^+^ MNP was also altered after *H. polygyrus* infection. A specific and significant increase in the number of CD11c^+^ MNP within the B cell mantle region of the Peyer’s patches on d 8 after *H. polygyrus* infection was observed (Fig. 8E). The distribution of the CD68^+^ MNP, in contrast, was unchanged (Fig. 8F) consistent with the cell abundance data (Fig. 8C). This analysis suggested that the significantly disturbed distribution of the MNP within the Peyer’s patches on d 8 after *H. polygyrus* infection may have impeded the ability of these cells to efficiently propagate the prions from the SED region down towards the FDC within the B cell follicles.

## DISCUSSION

Factors that influence the early accumulation of prions in the small intestinal Peyer’s patches can directly influence oral prion disease pathogenesis. Gastrointestinal pathogens can cause significant disturbances to the gut mucosa and the GALT. Therefore, in this study we determined whether pathology specifically in the small intestine, such as that caused by co-infection with the natural mouse helminth parasite *H. polygyrus* would influence oral prion disease pathogenesis. Mice were co-infected with prions at distinct intervals after *H. polygyrus* infection based on the parasite’s known pathogenesis in the host’s duodenum^39,44^ to determine whether the pathology in the duodenum during these distinct phases of the helminth infection affected oral prion disease pathogenesis. For example, we reasoned that damage to the epithelium might increase gut permeability, and by doing so raise the uptake of prions across it and exacerbate prion disease pathogenesis. Conversely, it was plausible that increased MNP abundance may enhance the sequestration of prions.

Our data revealed that the early accumulation of prions within the small intestinal Peyer’s patches was reduced and survival times significantly extended when the mice were co-infected with prions on d 8 after *H. polygyrus* infection. This effect was transient and specific to this period of the *H. polygyrus* infection, as no effects were observed when the mice were co-infected with prions at other intervals in relation to the helminth infection. This suggests that specific effects of the *H. polygyrus* co-infection on the Peyer’s patches had impeded the efficient spread of orally acquired prions from the gut lumen to the brain. We have previously shown that co-infection of mice with the large intestine-restricted helminth pathogen *Trichuris muris* does not influence oral prion disease pathogenesis^12^. This is most likely because the large intestinal GALT are not important early sites of prion accumulation and neuroinvasion^12^. In contrast, our data in the current study suggest that co-infections with small intestine dwelling helminth pathogens may be important factors that can significantly influence oral prion disease pathogenesis.

Little, if anything, was known of the effects of *H. polygyrus* infection on the key cell populations within the Peyer’s patches that play an important role in oral prion disease pathogenesis. M cells are considered to be the critical gate-keepers of oral prion infections^21,22^. However, the density or function of these cells was not affected by *H. polygyrus* infection. The FDC within the B cell follicles are the essential sites of prion replication in the GALT^12,52,53^, but the status of these cells was similarly unaffected in the Peyer’s patches of *H. polygyrus* infected mice. This clearly demonstrated that the effects of helminth infection on oral prion disease pathogenesis were independent of direct effects on M cells or FDC in Peyer’s patches.

Infections with gastrointestinal helminth parasites such as *H. polygyrus* characteristically induce robust T helper (Th) 2 immune responses associated with expression of cytokines such as interleukin (IL)-4 and IL-13, and potent regulatory T-cell responses^54^. However, data show that deficiency in T cells^55–58^ or the cytokines^59^ IL-4 or IL-13 do not influence peripheral prion disease pathogenesis or susceptibility. This suggests that the effects of helminth co-infection on oral prion disease pathogenesis were similarly independent of effects on T cells.

Following their initial transcytosis across the gut epithelium by M cells the prions are subsequently acquired by CD11c^+^ MNP such as the classical DC in the underlying SED regions of the Peyer’s patches^14,31,51^. The propagation of prions by these MNP towards the FDC in the Peyer’s patches is important to establish host infection and efficient neuroinvasion^32^. Our analysis revealed that the abundance of CD11c^+^ MNP in the B cell mantle region of the Peyer’s patches was specifically and significantly increased on d 8 after *H. polygyrus* infection. This implied that the disturbed distribution of the MNP within the Peyer’s patches on d 8 after *H. polygyrus* infection may have impeded the ability of these cells to efficiently propagate the prions from the SED region down towards the FDC within the B cell follicles. Other MNP populations such as macrophages appear to play a host protective role during prion disease by aiding the clearance of prions^30,33,34^. The “alternative” activation of macrophages is a characteristic feature of the helminth-induced Th2 response^60^. These alternatively-activated macrophages typically contribute to the regulation of inflammatory responses, and promote wound healing and parasite expulsion during helminth infections^39^. Whether effects of *H. polygyrus* infection on the activation status of the MNP in the Peyer’s patches may have also enhanced their ability to phagocytose and destroy the prions remains to be determined.

The factor/s during *H. polygyrus* infection that were responsible for the altered positioning and trafficking of the CD11c^+^ MNP within the Peyer’s patches are not known. Day 8 after *H. polygyrus* infection was selected as it is around this time that the adult worms emerge from the submucosa into the lumen of the duodenum^39,44^. During this process, significant amounts of parasite-derived antigens and debris from damaged host cells are liberated into the gut lumen. Thus, it is plausible that the presence of this material in the gut lumen stimulates the recruitment of CD11c^+^ MNP to the Peyer’s patches.

Orally-acquired prions accumulate first in the small intestinal Peyer’s patches and subsequently spread via the lymphatics and bloodstream to most other secondary lymphoid tissues, including the MLN and the spleen. However, our previous data show that the subsequent accumulation of the prions within these tissues is not essential for efficient neuroinvasion from the intestine^10,12,22,32^. In mice co-infected with prions on d 8 after *H. polygyrus* infection the accumulation of prions within the MLN was also reduced despite the presence of larger FDC networks. This suggests that any prions that are initially transported to the MLN immediately after oral exposure either reach this tissue in insufficient abundance to establish infection on FDC, or alternatively, are not propagated towards the B cell follicles. As a consequence, the prions are most likely phagocytosed and destroyed by the macrophages within the MLN^33,34^.

Studies of sheep with natural scrapie have revealed that orally acquired prions are similarly first detected in the GALT in the upper gastrointestinal tract before spreading to the draining lymph nodes and onwards to other lymphoid tissues. The large intestinal GALT such as the caecal patches are not early sites of prion accumulation^61–64^, consistent with data from experimental oral transmissions to mice^12^. Natural prion disease susceptible hosts such as sheep, deer and cattle are regularly exposed to helminths in field situations. Thus, co-infection with small intestine-restricted helminth pathogens around the time of oral exposure may similarly influence oral prion disease pathogenesis in naturally affected host species. An independent study has previously attempted to determine the influence that helminth infections may have on oral prion disease pathogenesis^65^. The gastrointestinal nematode parasite *T. circumcincta* establishes infection in the mucosa and lumen of the abomasum of sheep. When lambs with high genetic susceptibility to natural sheep scrapie were experimentally co-infected with *T. circumcincta* at monthly intervals from 6 to 11 months old, prion disease survival times were shortened^65^. However, very young lambs are highly susceptible to prion infection^66^ and are considered to acquire natural sheep scrapie from infected ewes via the oral route around the time of birth^67^. Treatments that can delay the accumulation of prions in the GALT do not influence disease pathogenesis once neuroinvasion has occurred and the prions have established infection in the peripheral nervous system^5^. Since the lambs in the above study^65^ were co-infected with *T. circumcincta* long after prion neuroinvasion from the intestine is likely to have occurred^61,62^, the reduced survival times are unlikely to be a consequence of effects of the parasite infection on the uptake or accumulation of the prions within the GALT. However, it is plausible that the parasite-mediated damage to the abomasum stimulated the systemic release of inflammatory mediators that exacerbated the development of neuropathology within the CNS^68^.

In the current study mice were given a single infection with *H. polygyrus*. Although experimental helminth infections in mice are commonly studied after administration of a single dose, natural host species in field situations are likely to be regularly exposed to these pathogens over long periods. The effects that chronic helminth exposure may have on MNP abundance and distribution within the Peyer’s patches are not known. It is plausible that repeated helminth exposure may lead to the prolonged recruitment of MNP into the B cell mantel region of the Peyer’s patches further enhancing the sequestration of orally-acquired prions.

Soft tissue granulomas are a prominent feature within chronically-inflamed tissues in humans and animals. One study has shown that an inflammatory PrP^C^-expressing stromal cell population in experimentally-induced soft tissue granulomas can support prion replication in infected mice^43^. Similar evidence of ectopic PrP^Sc^ accumulation has been observed in the newly formed lymphoid follicles of chronic lymphofollicular inflammatory sites in scrapie-affected sheep^69^. Granulomas can form around persistent foreign bodies, and are induced in the intestines of *H. polygyrus* infected mice in response to the invasion of the L3 larvae into the submucosa. However, the granulomas induced in the intestines of *H. polygyrus* infected mice predominantly contained MNP, lacked organized lymphoid follicles and PrP^C^-expressing stromal cells within them. Although the granulomas appeared to be surrounded by a border of PrP^C^-expressing stromal-like cells they were unable to support PrP^Sc^ accumulation in mice co-infected with prions.

Minor oral (lingual) lesions can enhance susceptibility to CWD prions in cervid PrP-expressing transgenic mice by facilitating direct neural invasion^26^. However, our own studies using two distinct gastrointestinal helminth pathogens show that the damage these parasites cause to the mucosa in the small (current study) and large intestines^12^ has little, if any, effect. Instead our data suggest that oral prion disease pathogenesis is significantly altered when the density or the status of the cells utilized by prions to establish infection within small intestinal Peyer’s patches is affected, including: M cells^15,21,22^; MNP^31,34^ (current study); and FDC^5,16^. Natural prion disease susceptible hosts such as sheep, deer and cattle are regularly exposed to helminths. Thus, co-infection with small intestine-restricted helminth pathogens around the time of oral prion exposure may be important factors that can influence prion disease pathogenesis in naturally affected host species.

## Materials and Methods

### Ethics Statement

Approvals for the mouse studies and regulatory licences were received from The Roslin Institute’s and University of Edinburgh’s ethics committees, and the UK Home Office. The animal experiments described in this study were undertaken under the authority of UK Home Office Project Licences (PPL60/4325 & PA75389E7) under the terms, conditions and regulations of the UK Home Office ‘Animals (scientific procedures) Act 1986’. Where appropriate and required, necessary efforts were undertaken to reduce harm and suffering. Mice were humanely culled at the end of the experiments using a UK Home Office Schedule One method.

### Mice

Six-eight weeks old female C57BL/6J (Charles River, Margate, UK) were used throughout this study. All mice were maintained under SPF conditions.

### Oral infection with *H. polygyrus*

Mice were orally infected by gavage with 200 *H. polygyrus* third stage (L3) larvae prepared as described^70^. Egg counts were determined by weighing faecal pellets from individual mice prior to resuspension in 1 ml of saturated sodium chloride/50% w/v glucose solution. Eggs were then counted using a McMaster chamber and the eggs/g faeces determined. For analysis of pathology, blinded sections of duodenum and ileum (2 per mouse) were scored for inflammatory cell infiltrate (0–4) and changes to the intestinal architecture (0–4) using the evaluation scheme described for *H. polygyrus* pathology in^41^. Mean scores per mouse were combined to give a maximum score of 8.

### Oral Prion Exposure and Disease Monitoring

Individual food pellets were first doused with 50 µl of scrapie brain homogenate derived from mice with terminal ME7 scrapie prion infection. Each pellet contained approximately 3.3 log_10_ intracerebral ID_50_ units. Each mouse was then individually housed in a bedding- and food-free cage with water provided *ad libitum*, and fed an individual prion doused food pellet^5,10,12,16,21,31^. The mice were returned to their original cages as soon as no signs of the food pellet were evident (indicating that it had been completely ingested). The mice were then coded and assessed by independent technicians at weekly intervals for the clinical signs of prion disease and scored as “unaffected”, “possibly affected” and “definitely affected” using standard criteria established for mice clinically-affected with ME7 scrapie prions^5,10,12,16,21,31^. Mice were either culled at the standard clinical endpoint, or survival times recorded for those that remained free of the clinical signs of prion disease after exposure. The clinical status in each mouse was confirmed by the histopathological assessment of prion disease pathology in the brain. The magnitude and distribution of the spongiform pathology (vacuolation) in nine distinct grey-matter regions of the brain was compared as described^71^.

### IHC and Immunofluorescent Analyses

To detect M cells by whole-mount immunostaining^21^ Peyer’s patches were first fixed using BD Cytofix/Cytoperm (BD Biosciences, Oxford, UK), and then immunostained with rat anti-mouse GP2 mAb (MBL International, Woburn, MA). The Peyer’s patches were then stained with Alexa Fluor 488-conjugated anti-rat IgG Ab (Invitrogen, Paisley, UK), rhodamine-conjugated *Ulex europaeus* agglutinin I (UEA-1; Vector Laboratories Inc., Burlingame, CA) and Alexa Fluor 647-conjugated phalloidin to detect F-actin (Invitrogen).

Intestines and MLN were snap-frozen at the temperature of liquid nitrogen, and 6 µm serial frozen sections cut using a cryostat. To detect FDC, sections were immunostained with anti-mouse CD35 mAb to detect CR1 (clone mAb 8C12; BD Biosciences, Wokingham, UK) and PrP-specific polyclonal antibody (pAb) 1B3 (ref.^72^) to detect cellular PrP^C^. To detect MNP, sections were immunostained with hamster anti-mouse CD11c mAb (clone N418, Bio-Rad, Kidlington, UK), rat anti-mouse CD11b mAb (clone M1/70; BioLegend, Cambridge, UK) or rat anti-mouse CD68 mAb (clone FA-11, BioLegend). Rabbit anti-synaptophysin 1 (Synaptic Systems, Göttingen, Germany) was used to detect nerve synapses. Sections were subsequently immunostained with streptavidin-conjugated or species-specific secondary antibodies coupled to Alexa Fluor 488 (green), Alexa Fluor 594 (red) or Alexa Fluor 647 (blue) dyes (Life Technologies). Cell nuclei were detected using DAPI (4′,6-Diamidine-2′-phenylindole; Fisher Scientific, Loughborough, UK). Sections were mounted in fluorescent mounting medium (DAKO) prior to imaging on a Zeiss LSM710 confocal microscope (Zeiss, Welwyn Garden City, UK).

Goblet cells were detected using a Periodic Acid Schiff (PAS) Stain Kit (Abcam, Cambridge, UK) according to the manufacturer’s instructions.

To detect disease-specific PrP (PrP^d^) in intestines, MLN and brains were first fixed in periodate-lysine-paraformaldehyde fixative and then embedded in paraffin wax and 6 µm sections cut. The sections were first pre-treated with a combination of hydrated autoclaving (15 min, 121 °C, hydration) followed by 5 min immersion in formic acid (98%) to enhance PrP^d^ detection, before immunostaining with 1B3 PrP-specific pAb. Astrocytes were detected by immunostaining with anti-glial fibrillary acidic protein (GFAP; DAKO, Ely, UK). Microglia were detect by pre-treatment with citrate buffer, followed by immunostaining with anti-ionized calcium-binding adaptor molecule 1 (Iba-1; Wako Chemicals GmbH, Neuss, Germany). To detect FDC, the sections were pre-treated with Target Retrieval Solution (DAKO) before immunostaining with rat anti-CD21/35 mAb (clone 7G6, BD Biosciences). Sections were counterstained with biotin-conjugated species-specific secondary antibodies (Stratech, Soham, UK) followed by HRP-conjugated to the avidin-biotin complex (ABC kit, Vector Laboratories, Peterborough, UK) with DAB (Sigma) as a substrate. Haematoxylin was used as a counterstain to detect cell nuclei. Paraffin-embedded tisssue immunoblot analysis^73^ was used to detect proteinase K-resistant PrP^Sc^ using with PrP-specific pAb 1B3.

### Image analysis

ImageJ software (http://rsb.info.nih.gov/ij/) was used for morphometric image analysis on coded sections as described^74^. First, pixel intensities across all the areas of each image (immunostained and non-stained regions) were measured using an ImageJ macro and used to obtain the background pixel intensity threshold values. These values were then applied to all subsequent analyses. The number of pixels in each colour channel (black, red, green, yellow etc.) in each image were then determined automatically. These data are presented as the proportion of positively immunostained pixels/total number of pixels (all colours) in the specific region of the image. We typically analysed 1–7 images from each mouse, and multiple mice were analysed from each group (*n = *3–8 mice/group). The figure legends display the sample sizes for every parameter analysed by this method in this study. For analysis of CD11c^+^ cell location, images were orientated with the FAE at facing upwards and the height of the patch and the Y-axis coordinate of each CD11c^+^ cell recorded. The location of each CD11c^+^ cell was calculated as a percentage of the total patch height and then allocated to three regions approximately representing the B cell follicle (0-33.33% of total patch height), B cell mantle (33.34–66.67%) and SED (66.68–100%).

### Oral gavage with fluorescent microbeads

Mice were given a single oral gavage of 2 × 10^11^ of Fluoresbrite Yellow Green labelled 200 nm microbeads (Polysciences, Eppelheim, Germany) in 200 µl PBS. Mice were culled 72 h later and Peyer’s patches and small intestine segments were snap-frozen at the temperature of liquid nitrogen. Serial frozen sections (6 µm in thickness) were cut on a cryostat and counterstained with DAPI. The number of beads in the SED from 3 sections of two Peyer’s Patches per mouse (*n* = 3–4 mice/group; total 4–19 SED/mouse studied) were counted. Images of SED were acquired using Nikon Eclipse E400 fluorescent microscope using Micro Manager (http://www.micro-manager.org). Tissue auto-fluorescence was subtracted from displayed images using ImageJ.

### Statistical Analyses

All data are presented as mean ± SD. Unless indicated otherwise, differences between groups were analysed by One- or Two-way ANOVA where appropriate. Comparisons between groups were performed using a Tukey’s (One-way ANOVA) or Sidak’s (Two-way ANOVA) post-hoc test. In instances where there was evidence of non-normality (identified by the Kolmogorov-Smirnov, D’Agostino & Pearson omnibus, or Shapiro-Wilk normality tests), data were analysed by appropriate non-parametric tests. Values of *P* < 0.05 were accepted as significant.

## Data Availability

All relevant data are present within the paper.
